# Flexible Ureteroscopy: Global User Experience Using Disposable Devices

**DOI:** 10.7759/cureus.46626

**Published:** 2023-10-07

**Authors:** Zain Kasmani, Manoj Ravindraanandan, Wasim Mahmalji

**Affiliations:** 1 Urology, Gloucestershire Hospitals NHS Trust, Cheltenham, GBR; 2 Urology, Wye Valley NHS Trust, Hereford, GBR

**Keywords:** environmental sustainability, technology development, renal stones, single-use flexible ureteroscope, flexible ureteroscope

## Abstract

Introduction: Renal stone treatment through flexible ureteroscopy is widely established and successful. Ureteroscopes can broadly be classified into reusable and single-use disposable devices, each with their own advantages. Disposable scopes are cheaper to buy, maintain, and dispose of but may have a greater environmental impact and long-term cost. To establish the collective views of urologists, we conducted a multicentre, global study to demonstrate users’ experience with single-use flexible ureteroscopes.

Methods: An online nine-question survey was distributed to urologists globally through email and social media platforms. Questions focused on user grade, experience, location, general opinion, advantages, disadvantages, and estimated cost of a single-use flexible ureteroscope. All responses were collated over a three-day period and analysed using descriptive statistics.

Results: A total of 69 responses were received; the majority of responses were from the UK (75%), and most were consultants (64%). Two-thirds of those surveyed had used a single-use scope on a patient, and 95% of them stated they enjoyed using it, citing excellent vision and reduced need for maintenance. The majority (52%) stated that widespread adoption of disposable scopes was limited due to their prohibitive expense, with an average, sterling-converted responder-estimated cost of £991 (£100-£6000) per reusable scope.

Conclusion: Most urologists enjoyed using disposable scopes, finding them comparable or better than reusable devices. However, the initial cost can be prohibitive in certain centres. The potential environmental impact is a further concern as this remains largely unknown for now. In the meantime, it is likely that stone units will continue to use a combination of single-use and reusable scopes, considering their individual needs and budgets as well as local availability and price.

## Introduction

Advancements in endourological instruments and improvements in laser technology have revolutionised the treatment for renal calculi [1]. Flexible ureteroscopy is, therefore, a mainstay of kidney stone treatment in many countries, with reports suggesting that they surpass other first-line treatment options by up to 30% [2]. Flexible ureteroscopy has become popular with urologists due to its high stone-free rate and patient acceptability when treating renal calculi [3,4].

Flexible ureteroscopes can be broadly divided into single-use, disposable devices or reusable scopes that require sterilising and maintenance but can be used for many years. Differing opinions and preferences exist when comparing these scopes. Many papers suggest that the initial cost of a reusable scope is prohibitively high. Adding this to the costs of sterilising, maintenance and repair caused by damage make these scopes unaffordable for some countries to sustain on a regular basis [1,5,6]. There are further issues of questionable durability and degradation secondary to repeated use, limiting uptake of reusable scopes due to the need for more frequent replacement or time out of use due to repair [7].

Previous studies have shown that single-use flexible ureteroscopes have possible superiority within these fields and that there may be a permanent role for them in the future [5]. We, therefore, devised a multi-centre study to demonstrate the collective view of users’ experiences with single-use flexible ureteroscopes around the world.

## Materials and methods

A questionnaire was created using the online platform SurveyMonkey [8]. This was subsequently distributed globally via email, personal contacts, and social media platforms to healthcare professionals working within urology. The data collected were anonymised. Responses were captured over a 72-hour period and later analysed using descriptive statistics.

There were a total of nine questions in the survey: five questions were multiple choice, and the remaining four allowed free-text answers (Table 1).

**Table 1:** Summary of questions within the SurveyMonkey questionnaire

Question	Multiple Choice (M)/Free Text (F)
What country do you work in? (List of countries)	M
What is the name of your hospital?	F
What is your designation within your hospital? (Senior House Officer, Registrar, Consultant, Other - specify)	M
Have you used a single-use ureteroscope on a patient before? (Yes/No)	M
If you have used a single-use ureteroscope before, did you like it? (Yes/No/NA)	M
Why did you like it?	F
In your opinion, what is stopping the widespread adoption of this surgical instrument? (Expensive, Environmentally unfriendly, Not better than reusable, Other - specify)	M
How much do you think a single-use ureteroscope costs?	F
Do you have any further comments?	F

## Results

Over a 72-hour period, we received 69 responses from urologists and colleagues within urology from around the world. Some answers were either not filled in by responders or intentionally left blank as it was not applicable to them.

Responders based in the UK made up 75.4% (n=54) of the total cohort, with the other 24.6% (n=15) coming from the rest of the world (Table 2).

**Table 2:** Global distribution of responders

Country	Number
England	49
Scotland	3
Wales	1
Northern Ireland	1
Republic of Ireland	1
Sweden	2
Italy	1
Spain	1
Australia	1
USA	1
Hong Kong	1
Malaysia	1
Singapore	1
Pakistan	1
India	1
Saudi Arabia	2
Chile	1

The majority of survey responders were at the consultant level 62.32% (n=43), 18.84% from urology specialist registrars (n=13), 8.7% (n=6) from urology trust grade doctors (associate specialists or senior clinical fellows), 5.8% (n=4) from urology senior house officers, and 4.35% (n=3) from other healthcare professionals.

Two-thirds of responders (66.67%, n=46) had said they had used a single-use flexible ureteroscope on a patient before, with 95.65% (n=44) saying that they liked using it.

**Figure 1:**
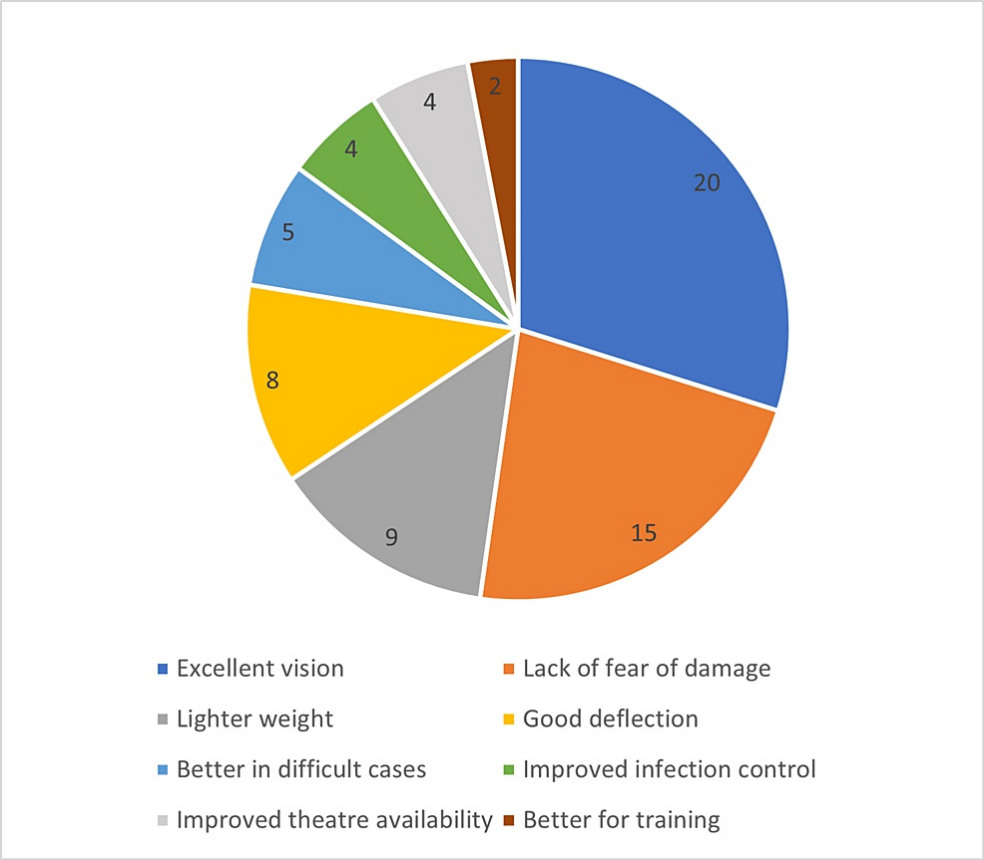
Reasons why responders liked the disposable flexible ureteroscope

For those who liked using a single-use flexible ureteroscope, reasons included (Figure 1): excellent vision (n=20), lack of fear of damage or need for maintenance (n=15), its lighter weight than a standard ureteroscope (n=9), good deflection (n=8), good for difficult or lower pole stones (n=5), better infection control (n=4), ease of availability in theatre (n=4), and useful for training trainees (n=2).

**Figure 2:**
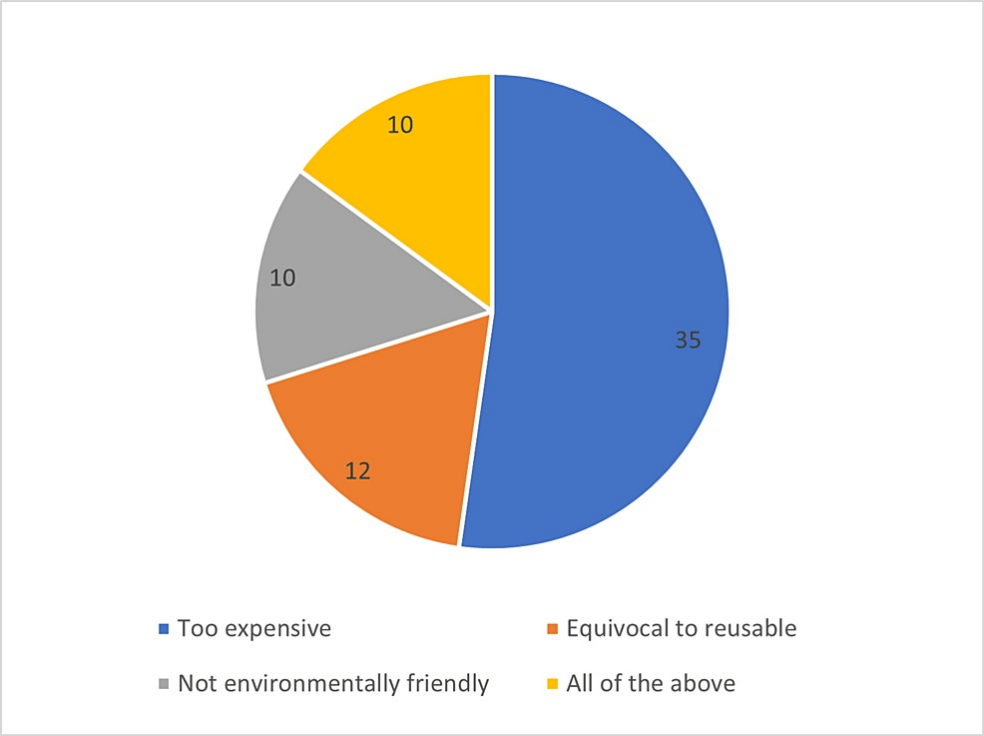
Reasons preventing the widespread adoption of single-use flexible ureteroscopes

When asked what was stopping the widespread adoption of a single-use flexible ureteroscope, 35 responders said that it was too expensive; 12 stated that they were not convinced that it is better than a traditional, reusable ureteroscope; 10 said that it was not environmentally friendly; and 10 mentioned all of the above (Figure 2).

When asked for a cost estimate of a single-use ureteroscope, after currency conversion to pound sterling, the estimated mean cost from responders was £991.36 with a wide range of £100-£6000.

## Discussion

Davis et al. have shown in their systematic review that single-use scopes have comparable patient outcomes to reusable scopes [9]. Our study adds to this by highlighting the overall global positive user experience whilst accepting that 75% of the views expressed are from UK clinicians. Excellent vision was quoted as the most common reason for the majority who liked using a disposable flexible ureteroscope. An in-vivo comparison of single-use to reusable scopes by Kam et al. has shown that the vision of two single-use scopes from different companies was close to the image quality of a reusable digital flexible ureteroscope - and in the future may even surpass it [10]. These findings were corroborated by Hennessey et al. [1] and Proietti et al. [5] who also mentioned that manoeuvrability and deflection were comparable to standard reusable scopes.

An issue with scope durability was a frequently seen response. Previous studies have shown damage with reusable scopes was a common occurrence, particularly with difficult cases or lower pole stones where great scope flexion is often required [1,6,11]. Furthermore, it was noted that, once damaged, the integrity of the scope was permanently compromised, leading to repeated damage on multiple occasions [7,11]. This ties in with the aforementioned comparable vision and deflection that a single-use scope provides, which nullifies future scope integrity due to their immediate disposable nature. Single-use scopes may, therefore, be preferable and more cost-effective in these complex cases.

The financial implication of a single-use ureteroscope was the biggest concern to the majority of responders. Multiple studies looking at the cost-benefit of a single-use scope have shown that, scope-for-scope, a reusable ureteroscope would provide higher savings when taking into account the risk of damage and repair associated with it, as well as the sterilising, cleaning, drying, and storage costs [6,10,12]. At the time of writing, studies have shown that, even though performance may be comparable, the cost of reusable scopes is far more economical compared to disposable scopes and that a significant reduction in price is needed to make single-use ureteroscopes a long-term viable option [12]. However, it has been mentioned that disposable scopes may have their place with selected cases of higher difficulty that would increase the chances of scope damage and, therefore, would save costs in the long term [6,9]. A separate cost-benefit analysis has shown that disposable scopes would be more economical for low-volume centres performing less than 50 cases a year, leaving it as a poor choice for high-volume specialist centres financially [9]. It was interesting to see the estimated costs for a single-use scope from responders with the range being from £100 to £6000. Estimates may be skewed as the cost of a single scope may vary from centre to centre, depending on their volumes or due to a lack of clarity provided by companies to urologists. Industry, and competition between providers, is certain to vary between regions and countries and, therefore, so will scope cost. Purchasing scopes in bulk, in countries where there is competition between providers, will work well for some centres but may not be feasible in less economically developed countries.

The environmental impact of disposable scopes was also mentioned in our survey. Given the rapid and life-changing implications our behaviours on recycling and waste in the past are having on the planet today, many are considering the implications single-use medical equipment is having on the environment. Efforts are being made to reuse or recycle equipment wherever possible. A study by Davis et al. compared the manufacturing, typical use, and disposal of both scopes and has shown that the carbon footprint per case left behind by a single-use ureteroscope was comparable to that of a reusable one (4.43 kg/CO_2_ for single use and 4.47 kg/CO_2_ for reusable) [13]. Of note, the washing and sterilisation of reusable scopes generate more carbon dioxide than the manufacturing of single-use scopes. There was no mention of the recycling of materials. Given that over 90% of the materials required to produce a scope come from plastic, there should be more emphasis on recycling and the impact it may have on the planet in the future. The recycling of single-use scopes may greatly increase their appeal, especially if coupled with much lower purchase costs.

## Conclusions

From our snapshot worldwide survey, we found that almost all urologists liked the single-use flexible ureteroscope. A single use-scope was superior in some categories: mainly vision, deflection, and a lack of fear of damage. By far, the biggest downside to a single-use ureteroscope is cost, followed by a potentially unfavourable environmental impact on the planet. However, with such a disparity in cost estimates from individuals, more education from manufacturers in the industry is needed to clarify and possibly change views. The cost of a single-use scope may vary from centres, depending on user volume so there may be a role in a ‘standardised’ single price. Accurate costs of sterilising, storing, and repairing reusable scopes have to be considered, as well as the environmental impact that this may have on the planet - which may be as detrimental as single-use scopes.

Scope comparability studies have been done in detail and shown patient outcomes are similar. Data from this study demonstrate that urologists worldwide like using single-use scopes. In-depth cost analyses are now required before a final decision can be made on whether single-use scopes should be adopted and in which settings. In the future, we feel that the modern stone unit would benefit from a combination of both reusable and single-use flexible ureteroscopes that can be used on a case-dependent basis. Urologists should familiarise themselves with the local costs of each scope, as well as the price of maintenance and repair. Using this information, they would be able to carefully select the best scope, or combination of scopes, that would be most suitable for their practice and patient requirements.
